# Activated Expression of Rice DMR6-like Gene *OsS3H* Partially Explores the Susceptibility to Bacterial Leaf Streak Mediated by Knock-Out *OsF3H04g*

**DOI:** 10.3390/ijms241713263

**Published:** 2023-08-26

**Authors:** Tao Wu, Yunya Bi, Yue Yu, Zhou Zhou, Bin Yuan, Xinhua Ding, Qingxia Zhang, Xiangsong Chen, Hong Yang, Haifeng Liu, Zhaohui Chu

**Affiliations:** 1 College of Plant Protection, Yangzhou University, Yangzhou 225009, China; taowu@yzu.edu.cn (T.W.); zqx817@126.com (Q.Z.); 2 State Key Laboratory of Hybrid Rice, Hubei Hongshan Laboratory, College of Life Sciences, Wuhan University, Wuhan 430072, China; yunyabi@163.com (Y.B.); sdauzbyy@163.com (Y.Y.); zhzhou20@whu.edu.cn (Z.Z.); chen.xs@whu.edu.cn (X.C.); 3 State Key Laboratory of Biocatalysis and Enzyme Engineering, School of Life Sciences, Hubei University, Wuhan 430062, China; yang@hubu.edu.cn; 4 Institute of Plant Protection and Soil Fertilizer, Hubei Academy of Agricultural Sciences, Wuhan 430064, China; yuanbin2000@139.com; 5 State Key Laboratory of Crop Biology, Shandong Agricultural University, Tai’an 271018, China; xhding@sdau.edu.cn (X.D.); hliu1987@sdau.edu.cn (H.L.)

**Keywords:** rice, bacterial leaf streak, DMR6-like, susceptibility, salicylic acid

## Abstract

Downy Mildew Resistance 6-like (DMR6-like) genes are identified as salicylic acid (SA) hydroxylases and negative regulators of plant immunity. Previously, we identified two rice DMR6-like genes, *OsF3H03g,* and *OsF3H04g*, that act as susceptible targets of transcription activator-like effectors (TALEs) from *Xanthomonas oryzae* pv. *oryzicola* (Xoc), which causes bacterial leaf streak (BLS) in rice. Furthermore, all four homologs of rice DMR6-like proteins were identified to predominantly carry the enzyme activity of SA 5-hydroxylase (S5H), negatively regulate rice broad-spectrum resistance, and cause the loss of function of these *OsDMR6s*, leading to increased resistance to rice blast and bacterial blight (BB). Here, we curiously found that an *OsF3H04g* knock-out mutant created by T-DNA insertion, *osf3h04g*, was remarkedly susceptible to BLS and BB and showed an extreme reduction in SA content. *OsF3H04g* knock-out rice lines produced by gene-editing were mildly susceptible to BLS and reduced content of SA. To explore the susceptibility mechanism in *OsF3H04g* loss-of-function rice lines, transcriptome sequencing revealed that another homolog, *OsS3H*, had induced expression in the loss-of-function *OsF3H04g* rice lines. Furthermore, we confirmed that a great induction of *OsS3H* downstream and genomically adjacent to *OsF3H04g* in *osf3h04g* was primarily related to the inserted T-DNA carrying quadruple enhancer elements of *35S*, while a slight induction was caused by an unknown mechanism in gene-editing lines. Then, we found that the overexpression of *OsS3H* increased rice susceptibility to BLS, while gene-editing mediated the loss-of-function *OsS3H* enhanced rice resistance to BLS. However, the knock-out of both *OsF3H04g* and *OsS3H* by gene-editing only neutralized rice resistance to BLS. Thus, we concluded that the knock-out of *OsF3H04g* activated the expression of the *OsS3H*, partially participating in the susceptibility to BLS in rice.

## 1. Introduction

One of the most important crops, rice, is widely cultivated in various regions, but it always suffers from attacks from various diseases with unfavorable climate and environmental conditions [1]. Salicylic acid (SA) is an important phytohormone in the regulation of plant defense against pathogens and abiotic factors [2,3]. In addition, the SA-mediated or -related defense response is one of the main components involved in resistance against diverse phytopathogens in rice. The nonexpressor of pathogenesis-related proteins 1 (NPR1) is the master regulator of the downstream SA signaling pathway and is recognized as an SA receptor to depolymerize into a monomer, which enters the nucleus to trigger defense-related gene expression. Other SA receptors, namely NPR3 and NPR4, are transcriptional repressors in SA-mediated defense [4,5]. Rice OsNH1, which functions as NPR1, is involved in the broad-spectrum defense against pathogens, and its mediated immunity requires the induction of cysteine-rich-receptor-like kinases gene *CRK10* by TGA transcription factor TGA2.1 [6,7]. Moreover, WRKY transcription factor OsWRKY45 plays an important role in SA-mediated defense against fungal and bacterial diseases, which are independent of OsNH1 [8,9,10]. In addition, the mutant of rice negative defense regulators usually results in an immune burst with an elevation of SA. Loss functions of several immune repressors, including mitogen-activated protein kinase OsMPK6 [11] and OsMPK15 [12], cytochrome P450 monooxygenase SL [13], C2H2-type transcription factor Bsr-d1 [14], and C2 domain Ca^2+^ sensor ROD1 [15], increase broad-spectrum resistance to various phytopathogens and the accumulation of SA content in rice.

In plants, SA biosynthesis is mainly related to the isochorismate and phenylalanine ammonia-lyase pathways [16]. The SA metabolism is composed of glycosylation, hydroxylation, methylation, sulfonation, and amino acid conjugations. The hydroxylation of SA into 2,5-dihydroxybenzoic acid (2,5-DHBA) and 2,3-dihydroxybenzoic acid (2,3-DHBA) by SA 5-hydroxylase (S5H) and SA 3-hydroxylase (S3H), respectively, is an inactive way to passivate plant immunity [17]. Previously, the loss function of DMR6/S5H, which encodes 2-oxoglutarate and Fe (II)-dependent dioxygenase (2OGDs), was found to confer resistance to downy mildew in *Arabidopsis* by the hydroxylation of SA into 2,5-DHBA [18,19,20]. In addition, S3H/DLO1, which is homologous to DMR6/S5H, was shown to possess the hydroxylation of SA into 2,3-DHBA in vivo of *Arabidopsis* [21]. Currently, several DMR6-like genes have been identified to confer resistance to phytopathogens after the loss of function in different plant species. The RNAi-mediated silencing of *StDMR6* and the gene-editing mediated knock-out of *StDMR6-1* conferred resistance to *Botrytis cinerea* and *Phytophthora infestans* in potatoes, respectively [22,23]. Similarly, the gene-editing of *SlDMR6-1* enhanced tomato broad-spectrum resistance to bacterial, oomycete, and fungal pathogens [24]. Moreover, the gene-editing of *ObDMR6* and *MusaDMR6* reduced susceptibility to *Peronospora belbahrii* and *Xanthomonas campestris* pv. *musacearum* in sweet basil and banana, respectively [25,26]. Recently, rice DMR6-like genes *OsF3H03g* and *OsF3H04g* were identified as the targets of transcription activator-like effectors (TALEs) Tal2b and Tal2c, respectively, which favor *Xanthomonas oryzae* pv. *oryzicola* (Xoc) infection in rice [27,28]. Subsequently, OsF3H03g (LOC_Os03g03034), OsF3H04g (LOC_Os04g49194), and the homolog of LOC_Os10g39140 were identified as S5H, while another homolog of LOC_Os04g49210 was identified as both of S5H and S3H. Notably, these genes negatively regulate SA-mediated broad-spectrum resistance in rice [29,30,31].

Xoc and *Xanthomonas oryzae* pv. *oryze* (Xoo) causes the main bacterial diseases, bacterial leaf streak (BLS) and bacterial blight (BB), respectively, in rice. They are also the model pathogens for plant–microbe interactions [32,33]. During the past few decades, over 42 BB resistance genes have been identified; however, few BLS resistance genes have been identified [34]. The first identified major BLS resistance gene, *Rxo1*, is a nucleotide-binding leucine-rich repeat (NLR) gene from non-host maize which confers resistance to Xoc through the recognition of non-TALE AvrRxo1 [35]. After that, *Xo1*, which contains the zinc finger BED and NLR domain from Carolina Gold Select rice, confers resistance to African Xoc strains, which is triggered by TALEs but does not rely on their ability of transcriptional activation [36,37,38]. A recessive resistance gene, *bls1*, encoding mitogen-activated protein kinase 6 (OsMAPK6), confers specific resistance to the Xoc strain JZ-8, while the overexpression of *BLS1* enhances rice broad-spectrum resistance [39,40]. Recently, *Xo2* characterized from Bangladeshi rice cultivar X455 was shown to confer resistance to most Chinese Xoc isolates, which involves fine mapping on chromosome 2 [41]. Mainly, the quantitative trait loci (QTLs) are responsible for rice BLS resistance [42,43,44,45]. Moreover, the constitutive expression of *OsMPK6* [46], *OsGH3-2* [47], *OsPGIP4* [48], *OsHSP18.0-CI* [49], *OsMAPK10.2* [50], *OsPSKR1* [51], *OsPGIP1* [52], *OsHSFB4d* [53], *OsPDX1.2* [54], and *OsCIPK15* [55] enhances rice resistance to BLS. The loss or depression of the function of *OsNRRB* [56], *OsImpα1a* and *OsImpα1b* [57], *OsALDH2B1* [58], *OsEDR1* [59], *OsAPS1* [60], *OsF3H03g* [28], and *OsNRAMP1* [61] increases BLS resistance in rice. On the other hand, gene-editing of the effector-binding element (EBE) in the promoter of *OsSULTR3;6*, which is a target of the major virulence factor Tal2g, confers rice resistance to multiple Xoc strains [62,63]. Interestingly, editing of EBE in the promoters of *OsF3H03g* or *OsF3H04g* confers rice-specific resistance to RS105/Tal2b and RS105/Tal2c, respectively [27].

Previously, we demonstrated that overexpressing the DMR6-like genes *OsF3H03g* and *OsF3H04g* reduced rice resistance to BLS and resulted in a decrease in SA content. Moreover, the gene-editing or suppressing of *OsF3H03g* conferred rice resistance to BLS [27,28]. Recently, studies have revealed that the gene-editing of *OsF3H04g* improves broad-spectrum resistance to rice blast and BB [29,31]. Unexpectedly, in this study, we identified an *OsF3H04g* T-DNA insertion mutant, *osf3h04g*, which was more susceptible to BLS and BB, as well as a tremendous reduction in SA content. However, the gene-editing mediated knock-out of *OsF3H04g* showed a slight increase in the susceptibility to BLS and a mild decrease in SA content. To explain the turnover functions of *osf3h04g*, transcriptome profiling was performed to reveal the activation of *OsS3H* by inserting T-DNA carrying quadruple enhancer elements that complemented the loss function of *OsF3H04g* and promoted the hydroxylation of SA to negatively regulate rice immunity. We revealed that the T-DNA mutant *osf3h04g* was composed of the knock-out of *OsF3H04g* and the constitutive activation of *OsS3H* mediated by the T-DNA carried enhancer elements, while the *OsF3H04g* loss function mutant induced a slight expression of *OsS3H* by an unknown mechanism. Furthermore, the knock-out of both *OsF3H04g* and *OsS3H* restored part of the rice resistance to BLS. Thus, we conclude that the loss function of *OsF3H04g* activates *OsS3H* to partially supplement the susceptibility to BLS in rice.

## 2. Results

### 2.1. T-DNA Insertion Line osf3h04g Is More Susceptible to BLS and BB

**Figure 1:**
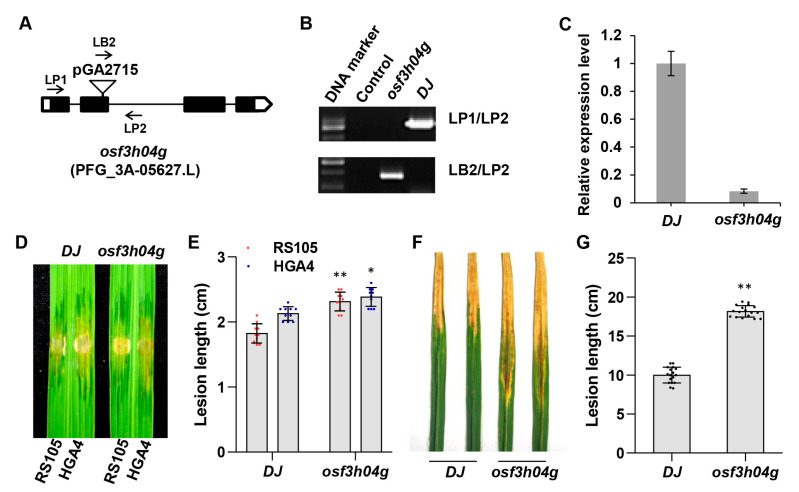
The identified T-DNA insertion line *osf3h04g* was susceptible to BLS. (**A**) Structure diagram for the T-DNA insertion in *osf3h04g*. (**B**) Identification of *OsF3H04g* T-DNA insertion rice *osf3h04g* by PCR amplification. The primer pairs LP1/LP2 and LB2/LP2 were used to amplify the genomic DNA fragment of *OsF3H04g* and the recombinant fragment of T-DNA and genome, respectively. (**C**) The expression level of *OsF3H04g* in *osf3h04g*. *OsACTIN* was used as a reference gene. Data represent means ± SD, n = 3. (**D**,**E**) Image of lesion expansions (**D**) and diagram of lesion lengths (**E**) in DJ and *osf3h04g* at 14 days post inoculation (dpi) with RS105 and HGA4. Data represent means ± SD, n = 10. (**F**,**G**) Phenotype of lesion expansions (**F**) and diagram of lesion lengths (**G**) in DJ and *osf3h04g* at 14 dpi with Xoo PXO99. Data represent means ± SD, n = 15. Asterisks indicate significant differences between DJ and *osf3h04g*. (* *p* ≤ 0.05; ** *p* ≤ 0.01, Student’s *t*-test).

Previously, we found that the overexpression of the DMR6-like gene *OsF3H03g,* which was targeted by Tal2b, could reduce rice resistance to Xoc and Xoo, while the loss-of-function could enhance the defense response of rice to the pathogens [28]. Subsequently, the overexpression of another DMR6-like gene, *OsF3H04g,* which is a target of Tal2c, resulted in increased susceptibility to Xoc [27]. However, the enhanced resistance to Xoc for the loss function of *OsF3H04g* is still unclear. To better understand the defense response involved with *OsF3H04g*, we obtained a T-DNA activation tagging line (PFG_3A-05627.L) from the rice T-DNA mutant library [64]. The homozygous mutant *osf3h04g* was identified as activation tagging vector pGA2715 inserted in the second exon of *OsF3H04g* (Figure 1A,B). Then, we performed qRT-PCR to reveal that *OsF3H04g* had decreased expression by a large margin in *osf3h04g* (Figure 1C). After that, we performed inoculation with the Xoc strains RS105 and HGA4 (hypervirulence strain containing Tal2b~Tal2e) on *osf3h04g*. As shown in Figure 1D, both RS105 and HGA4 caused more severe lesions in *osf3h04g* than in wild-type Dongjin (DJ). The lesion lengths were 2.32 ± 0.14 cm and 2.44 ± 0.17 cm in *osf3h04g*, which were longer than that of 1.82 ± 0.15 cm and 2.13 ± 0.10 cm in DJ caused by RS105 and HGA4, respectively (Figure 1E). Then, we also performed inoculation with Xoo strain PXO99 on DJ and *osf3h04g*. More severe disease symptoms and longer lesion lengths caused by PXO99 were also observed in *osf3h04g* than in DJ (Figure 1F,G). Thus, we revealed that the T-DNA insertion rice line *osf3h04g* is a null mutant and severely susceptible to BLS and BB.

### 2.2. Downregulated Defense-Related Genes and Salicylic Acid Content in osf3h04g

**Figure 2:**
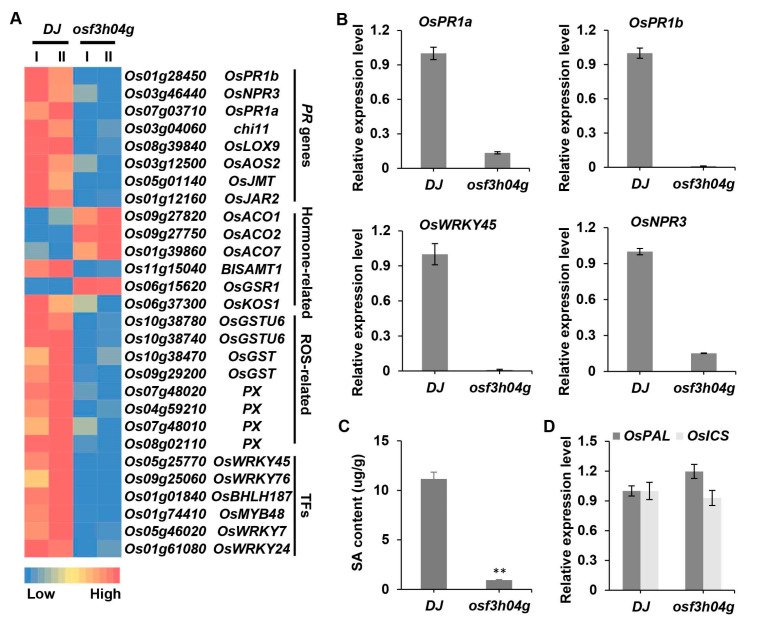
Defense-related and SA-related genes were suppressed, and extremely reduced salicylic acid content was observed in *osf3h04g*. (**A**) Heatmap of differentially expressed genes (DEGs) in correlation with pathogenesis-related (PR) genes, hormone-related genes, ROS-related genes, and transcription factors (TFs) in *osf3h04g*. I and II indicate the two independent replicates of RNA-seq. (**B**) The expression level of SA-related genes of *OsPR1a*, *OsPR1b*, *OsWRKY45,* and *OsNPR3* in *osf3h04g*. (**C**) Salicylic acid (SA) content in *osf3h04g*. (**D**) The expression level of *OsPAL* and *OsICS* in *osf3h04g*. *OsACTIN* was used as an internal control. Data represent means ± SD, n = 3. Asterisks indicate significant differences between DJ and *osf3h04g*. (** *p* ≤ 0.01, Student’s *t*-test).

To clarify that the regulation of gene expression in *osf3h04g* caused more susceptibility to BLS, we performed transcriptome sequencing for *osf3h04g* and wild-type DJ. The result showed that, in contrast with DJ, there were 826 differentially expressed genes (DEGs), which included 258 upregulated and 568 downregulated DEGs in *osf3h04g* (Table S1). After that, we performed a GO analysis of these DEGs and focused on those genes involved in the defense and stress response. The results showed that few genes were involved in defense response, and many genes were grouped into response to stress among the upregulated DEGs. However, there were several genes belonging to the GO terms of defense response, response to stress, and hormones, including the SA-related and JA-related response, among the downregulated DEGs (Figure S1). After that, we created a heatmap for these defense and stress response genes. The result showed that a considerable number of pathogenesis-related (*PR*) genes, ROS-related genes, and transcriptional factor genes showed repressed expression, while a few hormone-related genes, including ethylene- and gibberellin-related genes, had induced expression (Figure 2A). Among the downregulated DEGs in *osf3h04g*, we observed that a cluster of SA-related genes was downregulated (Figure 2A,B). *OsPR1a* and *OsPR1b* showed dramatically reduced expression in *osf3h04g* (Figure 2A,B). *OsWRKY45* and *OsNPR3,* which are involved in the SA-mediated signaling pathway, had significantly decreased expression in *osf3h04g* (Figure 2A,B). The abovementioned results indicate that the defense response and SA-related signaling pathway are alleviated in *osf3h04g*, which may relate to the reduced resistance to BLS and BB.

Previously, OsF3H04g was identified as an S5H as well as OsF3H03g and other homologs [29,30,31]. Overexpression of either *OsF3H04g* or any of the other three homologs could decrease SA content and SA-related genes expression, and the knock-out of any of the three homologs could accumulate SA content and activate broad-spectrum resistance [27,28,29,30,31]. Here, our results were different from those expected in *osf3h04g*, as SA-related genes had a suppressed expression as well as increased susceptibility. Then, we measured the content of SA in *osf3h04g*. The result showed that the SA content in *osf3h04g* was 0.94 ± 0.04 µg/g, which was dramatically reduced compared with that of 11.15 ± 0.68 µg/g in wild-type DJ (Figure 2C). To clarify whether the reduction in SA in *osf3h04g* was related to the synthesis, we then quantified the expression of the SA synthesis-related genes *OsPAL* and *OsICS*. However, the result showed that these two SA synthesis-related genes did not have reduced expression in *osf3h04g* (Figure 2D). Thus, the decreased SA content and normal expression of synthesis-related genes imply that other aspects of SA metabolism may be activated in *osf3h04g*.

### 2.3. Gene-Editing of OsF3H04g Increases Moderate Susceptibility to BLS in Rice

**Figure 3:**
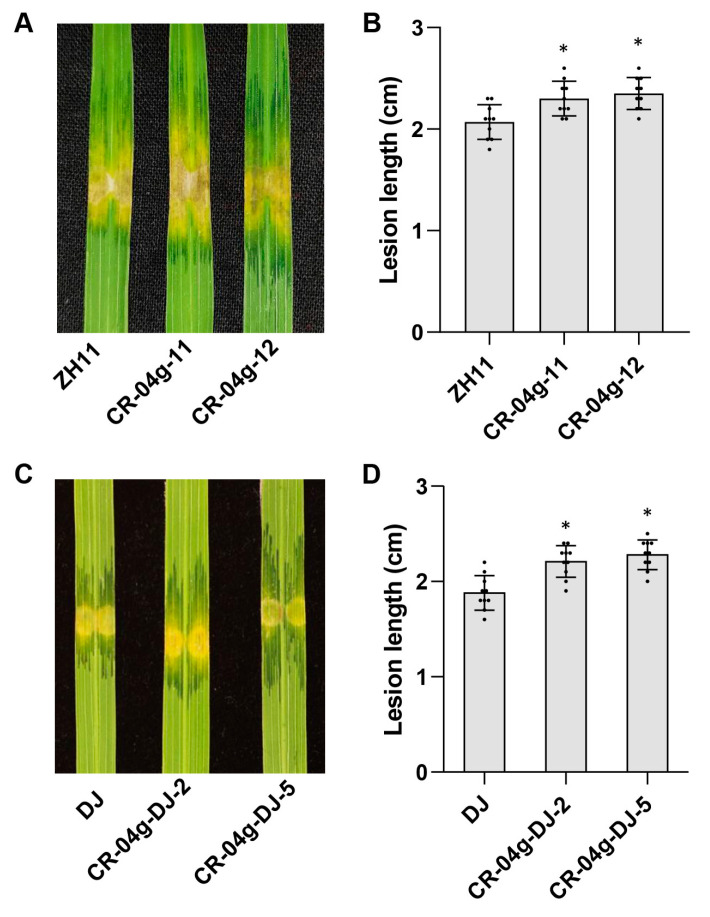
Gene-editing of *OsF3H04g* comprised rice resistance to BLS. (**A**,**B**) Image of lesion expansions (**A**) and diagram of lesion lengths (**B**) for the *OsF3H04g* gene-editing lines (CR-04g-11 and CR-04g-12) caused by Xoc RS105 at 14 dpi in ZH11. (**C**,**D**) Image of lesion expansions (**C**) and diagram of lesion lengths (**D**) in DJ for the *OsF3H04g* gene-editing lines (CR-04g-DJ-2 and CR-04g-DJ-5) inoculated with RS105 at 14 dpi. Data represent means ± SD, n = 10. Asterisks indicate significant differences between wild-type and *OsF3H04g* gene-editing lines. (* *p* ≤ 0.05, Student’s *t*-test).

The T-DNA line of *osf3h04g* showed more susceptibility to BLS and BB; however, our previous study revealed that the overexpression of this gene also increased rice susceptibility to BLS [27]. To clarify that the loss function of *OsF3H04g* is associated with increased susceptibility, we also generated the gene-editing lines of *OsF3H04g* mediated by the CRISPR-Cas9 system in the ZH11 background. Among the *OsF3H04g* gene-editing rice lines, only one type of mutation, a “T” base in the U3-gRNA target region and a “C” deletion in the U6a-gRNA target region were found in a total of eight T_0_ generation lines (Figure S2). Then, we randomly selected two lines of CR-04g-11 and CR-04g-12 to inoculate RS105 in T_1_ generation, which showed that the lesions in CR-04g-11 and CR-04g-12 developed slightly more rapidly than in wild-type ZH11 (Figure 3A). Furthermore, the lesion lengths were 2.30 ± 0.17 cm and 2.35 ± 0.16 cm in CR-04g-11 and CR-04g-12, respectively, which were mildly longer than that of 2.07 ± 0.17 cm in ZH11 (Figure 3B). After that, the SA content in the *OsF3H04g* gene-editing lines was also measured, which showed about 20 percent reductions in CR-04g-11 and CR-04g-12 compared with ZH11 (Figure S3). To validate the increased susceptibility, the gene-editing lines were also generated in the DJ background. A “T” base and a “C” base deletion in the U3-gRNA and U6a-gRNA target regions, respectively, were identified in CR-04g-DJ-2, while only a “C” base deletion in the U6a-gRNA target region was identified in CR-04g-DJ-5 (Figure S2). Similar to CR-04g-11 and CR-04g-12 in ZH11, CR-04g-DJ-2, and CR-04g-DJ-5 showed more susceptibility to RS105 with longer lesion lengths than that of DJ (Figure 3C,D). Interestingly, CR-04g-DJ-2 and CR-04g-DJ-5 also enhanced BB susceptibility to the Xoo strain of PXO99 (Figure S4). The abovementioned results indicate that gene-editing of *OsF3H04g* also increases rice susceptibility to BLS and decreases the SA level. Thus, our results demonstrate that the loss-of-function *OsF3H04g* compromises rice resistance to BLS.

### 2.4. RNA-Seq Analysis for Gene Expression Change in Loss-of-Function OsF3H04g Rice

**Figure 4:**
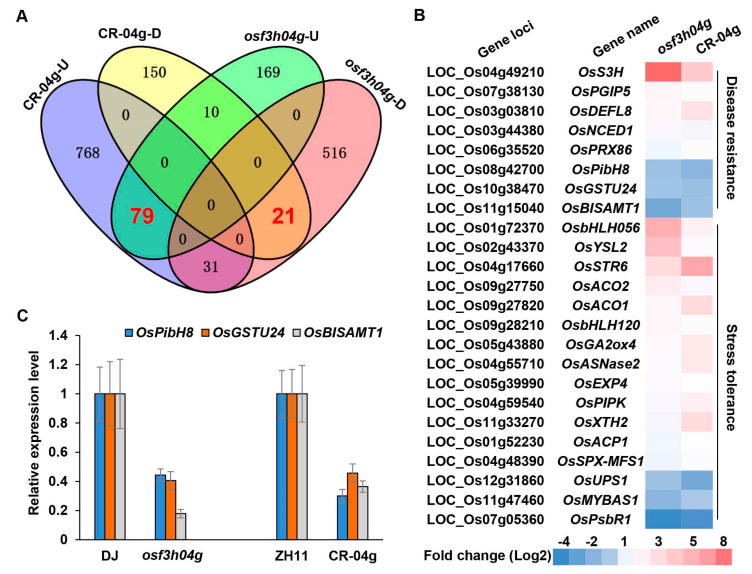
RNA-seq analysis for common differentially expressed genes in two loss-of-function *OsF3H04g* rice lines. (**A**) Venn diagram for the upregulated and downregulated DEGs in the *OsF3H04g* T-DNA line (*osf3h04g*) and gene-editing line (CR-04g-12). The number of the common DEGs between *osf3h04g* and CR-04g-12 is marked with bold red text. (**B**) Heatmap of the common DEGs involving disease resistance and stress resistance in *osf3h04g* and CR-04g-12. (**C**) The expression level of the common DEGs *OsBISAMT1*, *OsPibH8*, and *OsGSTU24* in *osf3h04g* and CR-04g-12. *OsACTIN* was used as an internal control. Data represent means ± SD, n = 3.

Here, we found that the *OsF3H04g* T-DNA line, as well as the gene-editing lines, increased rice susceptibility to BLS. The RNA-seq analysis revealed the defense response genes were seriously suppressed in the *OsF3H04g* T-DNA line. To reveal the differential regulation of defense response in the *OsF3H04g* gene-editing lines, we also performed the transcriptome sequencing for the *OsF3H04g* gene-editing line CR-04g-12 at the same condition of transcriptome sequencing for *OsF3H04g* overexpression line described in a previous report [27]. After comparing with wild-type ZH11, we found 878 upregulated and 181 downregulated DEGs in CR-04g-12 (Figure 4A, Table S2). Then, we compared these DEGs in CR-04g-12 with those identified in *osf3h04g*, which showed that 79 upregulated and 21 downregulated DEGs were common (Figure 4A, Table S3). After that, we performed the gene function analysis for these 100 common DEGs. The result showed that five upregulated and three downregulated DEGs were involved in disease resistance, while thirteen upregulated and three downregulated DEGs were related to stress tolerance (Figure 4B). Among these DEGs involved in disease resistance, a benzothiadiazole-induced rice blast resistance gene *OsPibH8* [65], a salicylic acid methyltransferase gene *OsBISAMT1* [66], and a glutathione S-transferase gene *OsGSTU24* [67] showed suppressed expression in *osf3h04g* and CR-04g-12 (Figure 4B,C). However, a DMR6-like gene, *OsS3H*, which is a negative regulator in rice broad-spectrum resistance [29,30], had highly induced expression in *osf3h04g* and mildly induced in CR-04g-12 (Figure 4B).

### 2.5. The OsS3H Is Activated Expression by the Adjacent Enhancer Elements in osf3h04g

**Figure 5:**
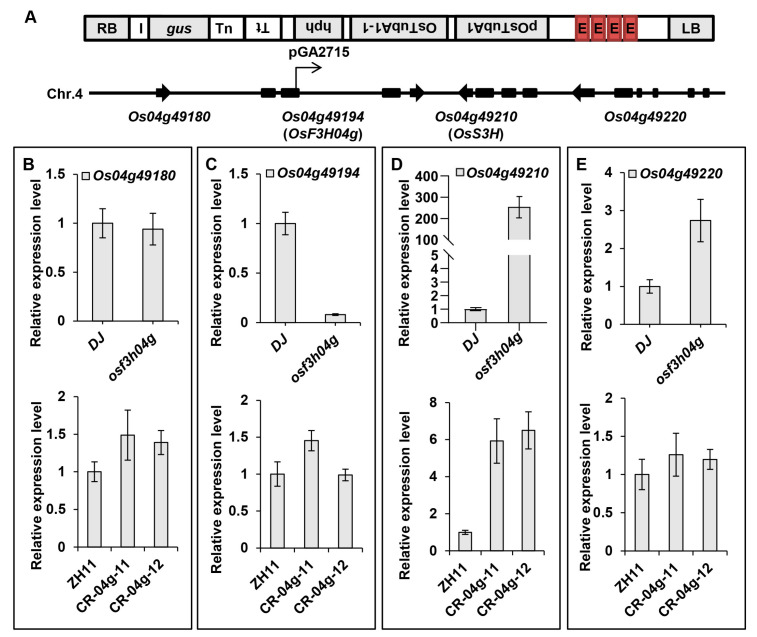
The *OsS3H* gene was activated expression by the T-DNA insertion in *osf3h04g*. (**A**) Structure diagram of the inserted T-DNA (pGA2715) and gene structure around T-DNA in the fourth chromosome of *osf3h04g*. Quadruple E indicates *35S* enhancer elements. (**B**–**E**) Expression level of *Os04g49180* (**B**), *Os04g49194* (*OsF3H04g*) (**C**), *Os04g49210* (*OsS3H*) (**D**), and *Os04g49220* (**E**) genomic adjacent to the T-DNA insertion position in *osf3h04g* and *OsF3H04g* gene-editing lines (CR-04g-11 and CR-04g-12). *OsACTIN* was used as an internal control. Data represent means ± SD, n = 3.

We found that the homologous *OsS3H* demonstrated a much more highly induced expression in the *OsF3H04g* T-DNA line than that in the gene-editing line. It was located downstream and genomic adjacent to *OsF3H04g* (Figure 5A). A previous report showed that the T-DNA vector, pGA2715, contained four enhancer elements of the *35S* promoter, which may affect the expression of genes upstream and downstream of the T-DNA inserted position [64]. Thus, we checked the expression levels of *Os04g49180*, *OsF3H04g* (*Os04g49194*), *OsS3H* (*Os04g49210*), and *Os04g49220,* which were close to the T-DNA in *osf3h04g* (Figure 5A). The result showed that the expression of *Os04g49180,* which was upstream of the T-DNA, was not changed significantly in *osf3h04g* or in the *OsF3H04g* gene-editing lines (Figure 5B). *OsF3H04g* had a dramatically decreased expression in *osf3h04g* compared to no significant change in expression in the ZH11 gene-editing lines of CR-04g-11 and CR-04g-12 (Figure 5C). Surprisingly, *OsS3H* activated a high expression of about 250-fold in *osf3h04g,* which was downstream of the T-DNA in *osf3h04g*, but only increased expression by several folds in CR-04g-11 and CR-04g-12 (Figure 5D). The *Os04g49220* gene, which was downstream of the T-DNA and *OsS3H,* showed about a three-fold induction in *osf3h04g* compared with DJ, and no significant change in expression was observed in the *OsF3H04g* gene-editing lines (Figure 5E). The abovementioned results indicate that a massive accumulation of *OsS3H* expression in *osf3h04g* is primarily caused by the quadruple *35S* enhancer elements, which may also relate to the enhanced susceptibility to BLS and reduction in the SA level.

### 2.6. OsS3H Plays a Negative Role in Rice Resistance to BLS and BB

**Figure 6:**
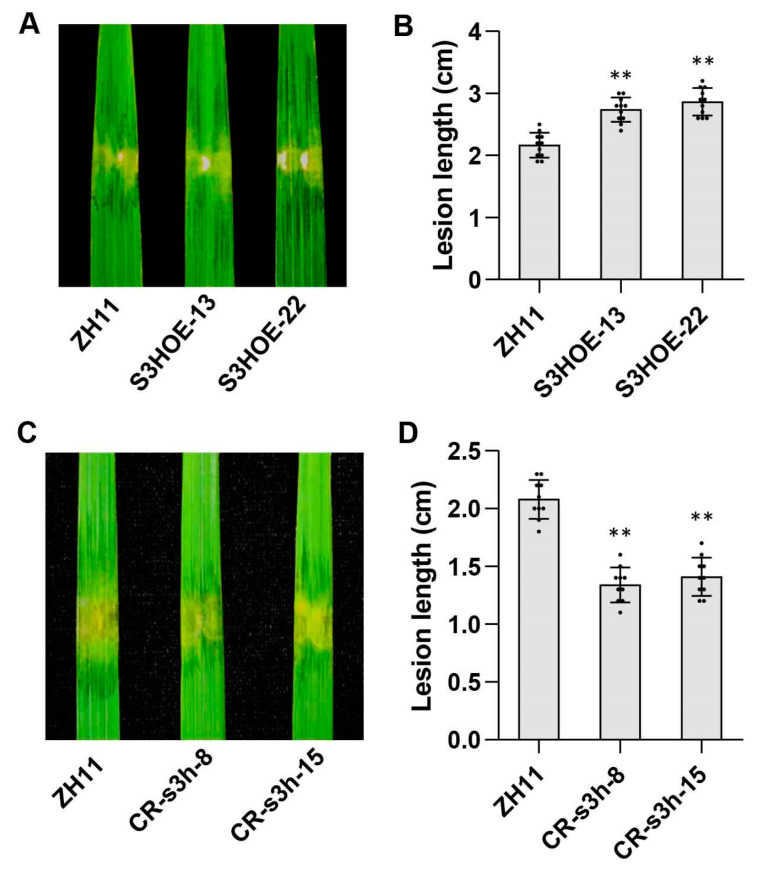
*OsS3H* negatively regulated rice resistance to BLS. (**A**,**B**) Image of lesion expansions (**A**) and diagram of lesion lengths (**B**) in ZH11 and the *OsS3H* OE lines (S3HOE-13 and S3HOE-22) caused by RS105 at 14 dpi. Data represent means ± SD, n = 10. Asterisks indicate significant differences between ZH11 and the *OsS3H* OE lines. (** *p* ≤ 0.01, Student’s *t*-test). (**C**,**D**) Image of lesion expansions (**C**) and diagram of lesion lengths (**D**) in ZH11 and the *OsS3H* gene-editing lines (CR-s3h-8 and CR-s3h-15) at 14 dpi with RS105. Data represent means ± SD, n = 10. Asterisks indicate significant differences between ZH11 and the *OsS3H* gene-editing lines. (** *p* ≤ 0.01, Student’s *t*-test).

The highly induced expression of *OsS3H* may be associated with increased susceptibility and decreased SA content in *osf3h04g*. Therefore, to clarify whether the induction of *OsS3H* is related to the susceptibility, we generated the *OsS3H* overexpression (OE) rice lines. Two *OsS3H* OE lines (S3HOE-13 and S3HOE-22) of 15 individual transgenic rice lines were selected for further study. After inoculation with RS105, we found that the lesion symptoms in *OsS3H* OE lines were more severe than that in the wild-type ZH11 (Figure 6A). As shown in Figure 6B, the lesion lengths in S3HOE-13 (2.74 ± 0.20 cm) and S3HOE-22 (2.86 ± 0.22 cm) were significantly longer than that of 2.16 ± 0.20 cm in ZH11. Furthermore, the increased lesion lengths were consistent with the constitutively enhanced expression of *OsS3H* in S3HOE-13 and S3HOE-22 (Figure S5). After that, we also performed inoculation with Xoo strain PXO99 in *OsS3H* OE lines. The result showed that longer lesion lengths caused by PXO99 were observed in S3HOE-13 and S3HOE-22 than those in ZH11 (Figure S6A,B). In addition, we also measured the SA content in the *OsS3H* OE lines. Compared with ZH11, the SA content was decreased by over 50 percent in both S3HOE-13 and S3HOE-22 (Figure S7). We then quantified the gene expression of *OsPR1b*, *OsNPR3*, and *OsWRKY45* in these lines, which showed that these SA-related genes had decreased expression in S3HOE-13 and S3HOE-22 compared with ZH11 (Figure S8A). The abovementioned results demonstrate that the overexpression of *OsS3H* results in a reduction in SA content and more susceptibility to BLS and BB in rice.

We also performed the gene-editing of *OsS3H* in ZH11 rice. Among the *OsS3H* gene-editing lines, two of them were identified as an “A” base and as a “T” base inserted in the genome of CR-s3h-8 and CR-s3h-15, respectively (Figure S9). Then, we performed inoculation with RS105 in CR-s3h-8 and CR-s3h-15. The results showed that the symptoms in CR-s3h-8 and CR-s3h-15 developed more slowly than that in ZH11 (Figure 6C). The lesion lengths were 1.34 ± 0.15 cm and 1.41 ± 0.14 cm in CR-s3h-8 and CR-s3h-15, respectively, which were apparently shorter than that of 2.08 ± 0.17 cm in ZH11 (Figure 6D). Furthermore, we also performed inoculation with PXO99 in these gene-editing lines. Compared with ZH11, lighter disease symptoms and shorter lesion lengths in CR-s3h-8 and CR-s3h-15 were observed (Figure S6C,D). Then, we also performed the quantification of SA content in the *OsS3H* gene-editing lines. The results showed that a significantly increased SA level was displayed in CR-s3h-8 and CR-s3h-15 compared with ZH11 (Figure S7). After that, the SA-related genes were examined in terms of expression level in the *OsS3H* gene-editing lines. The expression levels of *OsPR1b*, *OsNPR3*, and *OsWRKY45* were all enhanced several times in CR-s3h-8 and CR-s3h-15 compared to those in ZH11 (Figure S8B). Thus, our results indicate that *OsS3H* plays a negative role in the regulation of SA content and the defense response to BLS and BB in rice.

### 2.7. Gene-Editing Both OsF3H04g and OsS3H Neutralizes Rice Resistance to BLS

**Figure 7:**
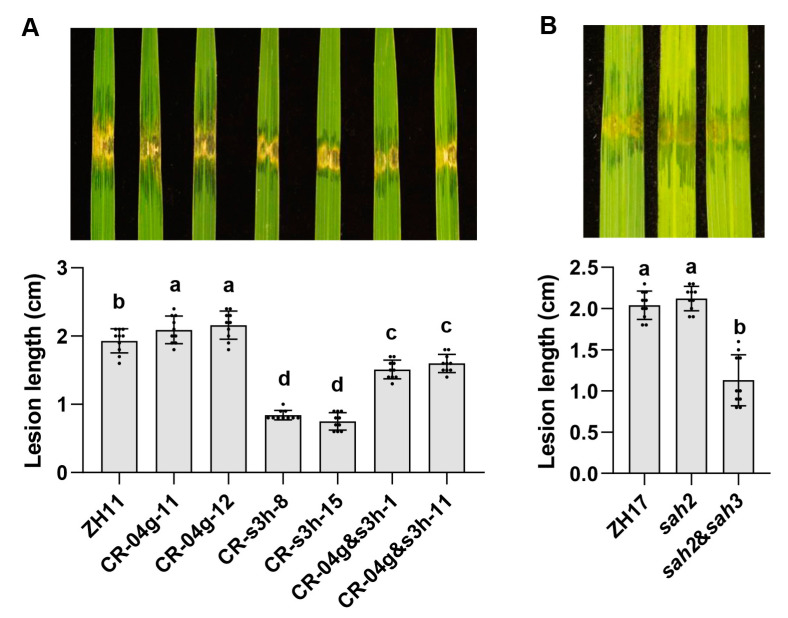
Gene-editing of *OsS3H* neutralized the rice susceptible to BLS mediated by the knock-out of *OsF3H04g*. (**A**) Image of lesion expansions and diagram of lesion lengths in ZH11, *OsF3H04g* gene-editing lines (CR-04g-11 and CR-04g-12), *OsS3H* gene-editing lines (CR-s3h-8 and CR-s3h-15), and *OsF3H04g* and *OsS3H* double gene-editing lines (CR-04g&s3h-1 and CR-04g&s3h-11) in the ZH11 background at 14 dpi with RS105. (**B**) Image of lesion expansions and diagram of lesion lengths in the *OsF3H04g*/*OsSAH2* gene-editing line (*sah2*), *OsF3H04g*/*OsSAH2,* and *OsS3H*/*OsSAH3* double gene-editing line (*sah2*&*sah3*) in the ZH17 background at 14 dpi with RS105. Data represent means ± SD, n = 10. The letters on the columns mean significant differences at a value of *p* ≤ 0.05 (LSD test).

The gene-editing of *OsF3H04g* showed increased susceptibility to BLS and induced expression of *OsS3H*; however, the overexpression of *OsS3H* reduced rice resistance, and the gene-editing of *OsS3H* resulted in increased rice resistance. To verify whether the induction of *OsS3H* expression in the *OsF3H04g* gene-editing rice line was related to the increased susceptibility, we generated the double gene-editing line of *OsF3H04g* and *OsS3H* rice in the ZH11 background. Two *OsF3H04g* and *OsS3H* double gene-editing lines named CR-04g&s3h-1 and CR-04g&s3h-11 were both identified as a “T” base deletion in the U3-gRNA target region in the *OsF3H04g* genome, and as a “T” base insertion in the U6a-gRNA target region in the *OsS3H* genome (Figure S10). Then, we simultaneously performed inoculation with RS105 in CR-04g&s3h-1 and CR-04g&s3h-11, as well as in the *OsF3H04g* or *OsS3H* single gene-editing lines as a parallel. The results showed that the lesion extensions and lesion lengths in CR-04g-11 and CR-04g-12 were more rapid and longer than that in wild-type ZH11, and CR-s3h-8 and CR-s3h-15 were significantly slower and shorter than that in ZH11 as described above; however, the lesion extension and lesion lengths in CR-04g&s3h-1 and CR-04g&s3h-11 were in an intermediate level between the *OsF3H04g* and *OsS3H* single gene-editing lines but still slower and shorter than that in ZH11 (Figure 7A). Thus, the abovementioned results indicate that the double knock-out of *OsF3H04g* and *OsS3H* neutralizes the increased susceptibility to BLS for the *OsF3H04g* single gene-editing line.

A previous study showed that the gene-editing of *OsF3H04g*/*OsSAH2* and *OsS3H*/*OsSAH3* increased resistance to rice blast, sheath blight, and BB in the ZH17 background [29]. Here, we also performed inoculation with Xoc strain RS105 in the *OsF3H04g/OsSAH2* single gene-editing line, as well as the *OsF3H04g/OsSAH2* and *OsS3H/OsSAH3* double gene-editing line in the ZH17 background, which showed that the gene-editing of *OsF3H04g/OsSAH2* also slightly increased susceptibility to BLS, while the gene-editing of *OsF3H04g*/*OsSAH2* and *OsS3H*/*OsSAH3* markedly increased resistance to BLS (Figure 7B). Then, we performed inoculation with PXO99 in the *OsF3H04g* and *OsS3H* double gene-editing lines in the ZH11 background. The result showed that the gene-editing of *OsF3H04g* in the ZH11 background had no significant effect on rice resistance to BB, while the double gene-editing of *OsF3H04g* and *OsS3H* increased rice resistance to BB, and the degree of increased resistance in the double gene-editing lines was still less than that in *OsS3H* gene-editing lines (Figure S11). Our results indicate that the gene-editing of *OsF3H04g* in ZH11 and ZH17 rice varieties increases susceptibility to BLS, which could be neutralized by the knock-out of *OsS3H*.

## 3. Discussion

DMR6 and DMR6-like genes were previously found as downy mildew susceptibility genes in *Arabidopsis* [18,19]. They usually function as SA hydroxylase to repress SA-mediated defense [20,21]. Recently, several DMR6-like genes have been identified in potatoes [23], tomato [24], sweet basil [25], banana [26], grapevine [68], and rice [27,28,29,30,31], that enhance resistance to pathogens after the loss of function in corresponding plants. In this study, we identified the loss function of a rice DMR6-like gene *OsF3H04g* for either T-DNA insertion or gene-editing, which unexpectedly resulted in increased susceptibility to BLS. However, the *OsF3H04g* T-DNA activation tagging line showed increased susceptibility to BLS and extremely decreased SA content compared to that of the gene-editing rice lines. We also revealed that another DMR6-like gene, *OsS3H*, located downstream of *OsF3H04g*, showed a highly induced expression in the T-DNA line, which was mainly caused by the insertion of the quadruple enhancer elements in the T-DNA sequence. *OsS3H* also has a slightly induced expression in the gene-editing lines by an unknown regulation. We then confirmed that *OsS3H* had a negative role in rice defense against Xoc, and gene-editing of both genes only partially recovered rice resistance to BLS. Consequently, our results revealed an abnormal case of the DMR6-like gene in rice susceptible to BLS, of which the loss of function could regulate the expression of a genomic downstream homolog to supplement the SA metabolism.

Recent studies have shown that the gene-editing of *OsF3H04g/OsSAH2*/*OsS5H2* increased resistance to BB and blast in rice varieties ZH17 and Nipponbare [29,31]. However, in this study, we revealed that the loss function of this gene increased susceptibility to BLS in rice varieties DJ and ZH11 (Figure 1 and Figure 3). We also found that the gene-editing of *OsSAH2* in the ZH17 background slightly increased susceptibility to BLS (Figure 7). In addition, the *OsF3H04g* T-DNA line in the DJ background was more susceptible to BB, which may predominantly relate to a great induction of *OsS3H*. The *OsF3H04g* gene-editing lines of DJ also showed increased susceptibility to BB, while the *OsF3H04g* gene-editing lines of ZH11 showed no significant effect on susceptibility to BB (Figure 1 and Figures S4 and S11). Thus, we propose that the gene-editing of *OsF3H04g* has a distinct role in rice defense against BLS and BB, as well as a diversity of susceptibility to BB in different rice backgrounds. We observed that the backgrounds of rice varieties used for the generation of *OsF3H04g* knock-out lines are different. Comparing analyses from two recent publications, the rice variety Nipponbare is more resistant to Xoo PXO99, causing a shorter lesion length than ZH17 [29,31]. We observed that the gene-editing of *OsF3H04g* in Nipponbare enhanced resistance to BB more than rice variety ZH17 [29,31]. Also, the different *M*. *oryzae* strains used in the two studies showed minutely increased resistance to rice blast compared with the highly increased resistance in the ZH17 background [29,31]. Herein, the gene-editing of *OsF3H04g* in ZH11 showed reduced resistance to BLS, while no significant impact on resistance to BB was observed (Figure 3 and Figure S11). Thus, we consider that the loss-of-function *OsF3H04g* in different rice backgrounds has diverse roles in rice resistance to different pathogens.

The T-DNA vector pGA2715 carries quadruple cauliflower mosaic virus *35S* enhancer elements, which are used for the generation of activation tagging lines. Usually, the insertion caused by this vector activates the expression of upstream or downstream genes flanking the insertion site in rice [64,69]. Here, we considered that the T-DNA activation tagging line *osf3h04g* was different from gene-editing lines. It is easy to understand that the insertion of the enhancer traps the knock-out of *OsF3H04g* and boosts the expression of *OsS3H* to accelerate the SA metabolism. The results showed that *OsS3H* induced hundreds of folds in *osf3h04g* (Figure 5). The SA level was reduced more than 10 times in *osf3h04g* and was reduced by about half in the *OsS3H* overexpression lines (Figure 2 and Figure S4). However, we found that *OsS3H* had a slightly induced expression in the *OsF3H04g* gene-editing lines (Figure 5D), as well as a slightly induced expression compared to that previously reported in ZH17 [29]. Consistent with the results, the slightly induced expression of *OsS3H* decreased about 30 percent of the SA content in the *OsF3H04g* gene-editing lines (Figure 2 and Figure S4). Alternatively, the *PR* genes, including *OsPR1b*, *OsNPR3,* and *OsWRKY45*, were downregulated in the *osf3h04g* and *OsS3H* overexpression lines but not in the *OsF3H04g* gene-editing line (Figure 2 and Figure S7). Thus, we concluded that there are two ways to increase *OsS3H* expression: the enhancer trapping induction and an unidentified mechanism that slightly induces the expression. We speculate that the slight induction of *OsS3H* in the loss-of-function *OsF3H04g* rice lines may relate to the feedback of the elevated SA level when one of the SA hydroxylases is broken one explanation, and the endogenous transcriptional regulation of these SA hydroxylase family genes to balance SA metabolism in rice may be another explanation.

Rice contains four DMR6-like genes, which include *OsF3H03g*/*OsSAH1*/*OsS5H2* (*LOC_Os03g03034*), *OsF3H04g*/*OsSAH2*/*OsS5H3* (*LOC_Os04g49194*), *OsS3H*/*OsSAH3*/*OsS5H1* (*LOC_Os04g49210*), and *OsSAH4*/*OsS5H4* (*LOC_Os10g39140*). OsF3H03g and OsS3H, the main S5H in rice, are negative regulators in the SA-mediated defense response of rice to diverse pathogens [27,28,29,30,31]. Previously, we reported that the overexpression of *OsF3H04g* decreased SA content and increased susceptibility to BLS [27]. However, the loss-of-function *OsF3H04g* also resulted in increased susceptibility to BLS in this study (Figure 1 and Figure 3). Interestingly, in the *OsF3H04g* gene-editing lines, *OsS3H* showed an increased expression. Moreover, we also confirmed that the overexpression of *OsS3H* increased rice susceptibility to BLS in wild-type ZH11 rice. We found that the gene-editing of *OsS3H* increased rice resistance to BLS; however, the gene-editing both of *OsF3H04g* and *OsS3H* did not recover the whole resistance to BLS, nor did the *OsS3H* gene-editing lines (Figure 7). This may indicate that the induction of *OsS3H* in the *OsF3H04g* gene-editing lines only partially contributes to the BLS susceptibility. There may be alternative enhanced susceptibility mechanisms to be identified in the future. On the other hand, we observed that chemicals and *M*. *grisea* response genes, *OsPibH8* [65] and *OsBISAMT* [66], were downregulated in the loss function of the *OsF3H04g* lines. Several hormone-related genes, including abscisic acid-related genes *OsNCED1*, *OsPsbR1*, and *OsbHLH120*; ethylene-related genes *OsACO1* and *OsACO2*; and gibberellin-related genes *OsGA2ox4*, *OsYSL2*, and *OsMYBAS1*, commonly showed changed expressions (Figure 4). In addition, most of the common DEGs in the loss function of *OsF3H04g* lines had an unknown function. Future work may focus on the other components participating in BLS susceptibility mediated by the loss-of-function *OsF3H04g* in rice.

## 4. Materials and Methods

### 4.1. Plant Materials

The *OsF3H04g* T-DNA line, *osf3h04g* (PFG_3A-05627.L), was inserted with an activation tagging vector pGA2715 in *Oryza sativa* L. ssp. *Japonica* cv. DJ, which was obtained from the rice T-DNA insertion sequence database [64]. *Japonica* cv. ZH11 was used for generating CRISPR-cas9-mediated gene-editing and overexpression of rice lines. The *OsF3H04g*/*OsSAH2* gene-editing rice line *sah2*, *OsF3H04g*/*OsSAH2,* and *OsS3H*/*OsSAH3* double gene-editing rice line *sah2*&*sah3* in *Japonica* cv. ZH17 background was provided by Dr. Xujun Chen [29]. A growth chamber with a temperature of 28 ± 2 °C and a 14/10 h cycle of light/dark was used for rice cultivation. The rice lines, including wild-type and transgenic rice, were grown in the same pot filled with soil.

### 4.2. Pathogen Inoculation

The Xoc strains RS105 and HGA4 were used for BLS disease measurement, and the Xoo strain PXO99 was used for BB disease evaluation, according to a previous report [28]. Briefly, the OD600 = 0.5 bacterial cell cultures of Xoc and Xoo strains were resuspended in sterile water, and inoculation was performed by injection with a non-needle syringe and the leaf-clipping method, respectively. The rice growth at 6 weeks after the seeding and booting stage was used to perform inoculation with Xoc and Xoo, respectively.

### 4.3. DNA Manipulation

Total plant genomic DNA was isolated through CTAB, as described in a previous report [70]. The genomic DNA of *osf3h04g* was used for amplification with specific primer pairs provided by SIGnAL-RiceGE (http://signal.salk.edu/cgi-bin/RiceGE, accessed on 10 June 2015) to identify the homozygous gene. To identify the *OsF3H04g* and *OsS3H* gene-editing lines, the fragments, including the gRNA target sites, were amplified with specific primers from the genomic DNA of the gene-editing lines. The primers are listed in Table S4.

### 4.4. RNA-seq and qRT-PCR

According to a previous report [52], 8-week-old rice leaves of DJ and *osf3h04g* were collected to isolate total RNA by TRIzol reagent. Two individual RNA repeats of each rice line were subjected to library construction and RNA sequencing by BGISEQ 500 (Beijing Genomics Institution, Shenzhen, China). The accession number of PRJNA983096 for the raw sequence reads was submitted to the NCBI Sequence Read Archive (SRA). The significant DEGs between DJ and *osf3h04g* were determined by absolute log2-ratio values ≥ 1 and *p* ≤ 0.05. Gene ontology (GO) analysis for the DEGs was implemented through the software of clusterProfiler 4.0. The above total RNA of DJ and *osf3h04g* rice were used as templates to generate cDNA by MonScript™ RTIII All-in-One Mix with dsDNase (Monad, Wuhan, China). Subsequently, the cDNAs were performed qRT-PCR with MonAmp™ SYBR^®^ Green qPCR Mix (Monad, Wuhan, China) by a CFX 96 system (BIO-RAD, Hercules, CA, USA). The primers used for qRT-PCR are listed in Table S4.

The RNA sequencing for the *OsF3H04g* gene-editing line (CR-04g-12) was performed with triple repeats as a parallel experiment with ZH11 and OsF3H04g overexpression lines according to the previous reports [27,28]. The accession number (PRJNA948698) of the raw sequence reads for CR-04g-12 was submitted to NCBI SRA. The DEGs were selected by the absolute log2-ratio values ≥ 1 and *p* ≤ 0.05 between CR-04g-12 and ZH11 (raw sequence reads of ZH11 were previously uploaded with the accession number PRJNA730674). The common and specific DEGs between *osf3h04g* and CR-04g-12 were identified by Venny 2.1 (https://bioinfogp.cnb.csic.es/tools/venny/index.html, accessed on 3 February 2021). The gene function analysis for the common DEGS was performed on Oryzabase (https://shigen.nig.ac.jp/rice/oryzabase, accessed on 22 February 2021). Expression level validation of the common DEGs was performed through qRT-PCR, as described above.

### 4.5. Gene-Editing and Overexpression Rice Construction

The gRNA target sequences, including U3 (5′-GCTCATCGACCTCGCCTCGC-3′) for *OsF3H04g*, were designed in E-CRISP (http://www.e-crisp.org/E-CRISP, accessed on 5 April 2018). The U3- and U6a-gRNA expression cassettes for *OsF3H04g* were cloned into the binary vector pYLCRISPR/Cas9-MH to generate pCas9-OsF3H04g according to the previous report [71]. To construct *OsS3H* overexpression rice, a 1059 bp CDS fragment of the *OsS3H* gene was amplified from ZH11 rice cDNA and then cloned into pCXUN-HA to generate the pUN-S3H vector. To construct *OsS3H* gene-editing rice, the U3 target sequence (5′-GCGCCGGCATTCCGGTCATC-3′) for *OsS3H* was designed in E-CRISP, and then the U3-gRNA expression cassette was formed to insert into pYLCRISPR/Cas9-MH to generate the pCas9-S3H vector. To construct *OsF3H04g* and *OsS3H* double gene-editing rice, the U3- and U6a-gRNA expression cassettes carried with target sequences for *OsF3H04g* and *OsS3H*, respectively, were connected and cloned into the pYLCRISPR/Cas9-MH to generate pCas9-F3H04g-S3H vector. The abovementioned vectors were individually transformed into *Agrobacterium tumefaciens* EHA105 by electroporation, and then the transformants were performed. The *A*. *tumefaciens*-mediated rice transgenic operation was performed according to a previous report [72].

### 4.6. Determination of Salicylic Acid

The *osf3h04g* and the wild-type DJ, *OsF3H04g* gene-editing lines (CR-04g-11 and CR-04g-12), and the wild-type ZH11 were grown for 6 weeks. Then, the leaves were collected for the quantitative analysis of SA content. The abovementioned tissue was ground into powder in surrounding liquid nitrogen, and 100 mg leaves powder per repeat, and three repeats per line were used for hormone extraction. The SA content was determined using the liquid chromatography-mass spectrometry (LC-MS) system (Waters, Milford, MA, USA), according to previous reports [27,28].

## 5. Conclusions

In this research, we revealed that the loss function of a rice DMR6-like gene, *OsF3H04g*, increased rice susceptibility to BLS and BB, which is in contrast to the results of previous studies. The loss of function of *OsF3H04g*, mediated by the insertion of enhancer elements or by gene-editing to enhance BLS susceptibility, was mainly or partially dependent on the activation of the expression of a downstream and genomically adjacent homolog *OsS3H*. Our research provides an exhaustive understanding of the rice DMR6-like family genes in resistance to different pathogens, which may provide guidance in breeding for broad-spectrum resistance in rice by selectively blocking the DMR6-like genes.

## Data Availability

Sequence data from this study can be found on the Rice Genome Annotation Project website (http://rice.plantbiology.msu.edu/, accessed on 13 February 2019) and NCBI (https://www.ncbi.nlm.nih.gov/, accessed on 7 December 2020) under the following accession number: *OsF3H04g* (*LOC_Os04g49494*), *OsS3H* (*LOC_Os04g49210*). Raw sequence reads of transcriptome sequencing for DJ, *osf3h04g*, and CR-04g-12 were performed in this study and uploaded to SRA to achieve the accession number (PRJNA983096 and PRJNA948698).

## Supplementary Materials

The following are available online at https://www.mdpi.com/article/10.3390/ijms241713263/s1.
