# Supplementary material for: Activated Expression of Rice DMR6-like Gene OsS3H Partially Explores the Susceptibility to Bacterial Leaf Streak Mediated by Knock-Out OsF3H04g
Source: Int J Mol Sci. 2023 Aug 26;24(17):13263. doi: 10.3390/ijms241713263 (PMC10487387; doi:10.3390/ijms241713263)
Supplement: Supplementary file 1 [file ijms-24-13263-s001.zip › 04g-IJMS-Supplementary.pdf]

Supplement figures

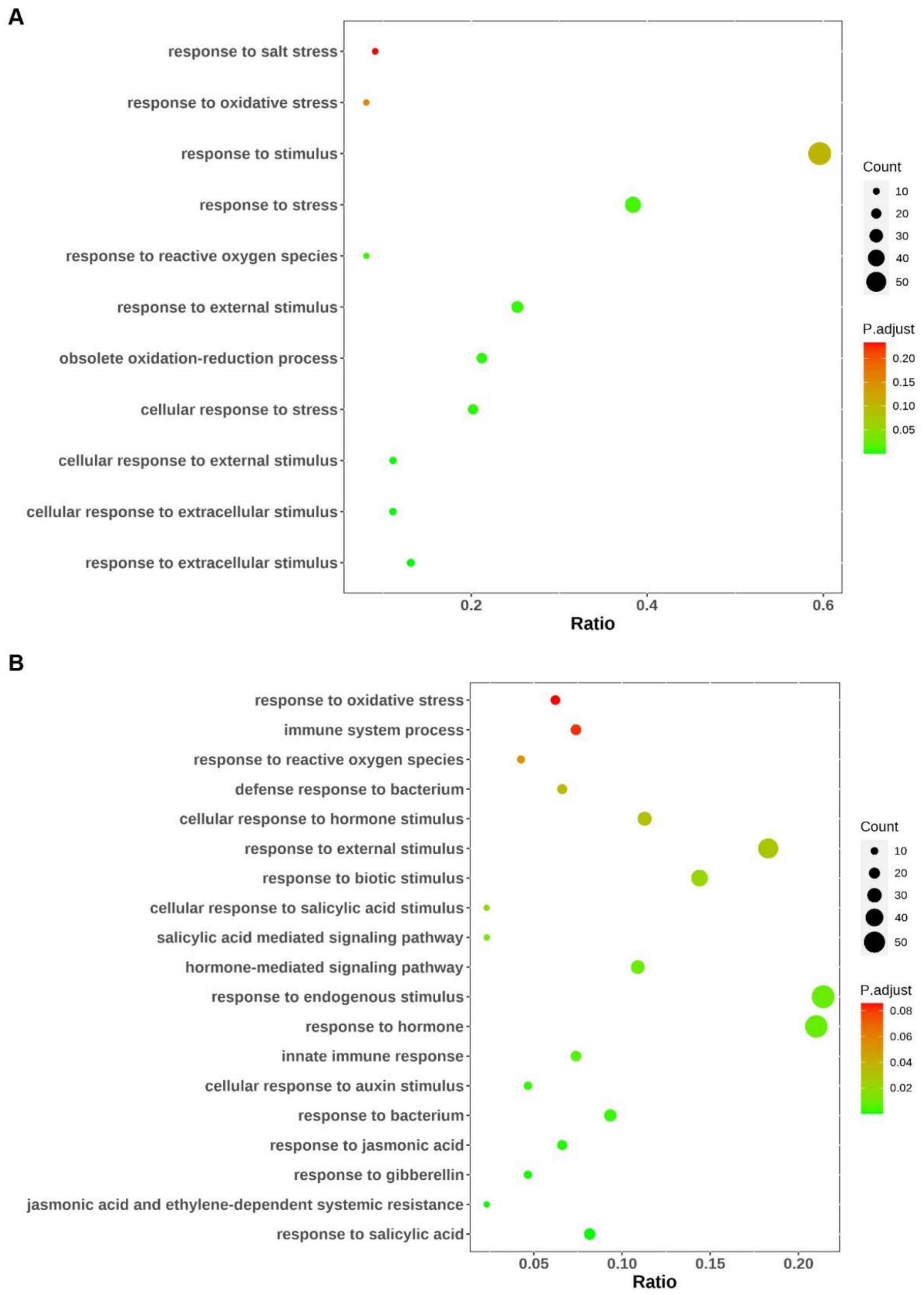

Figure S1. The gene ontology (GO) analysis of differentially expressed genes (DEGs) in *osf3h04g*. (A, B) The GO analysis of defense response for up-regulated DEGs (A) and down-regulated DEGs (B) in *osf3h04g*.

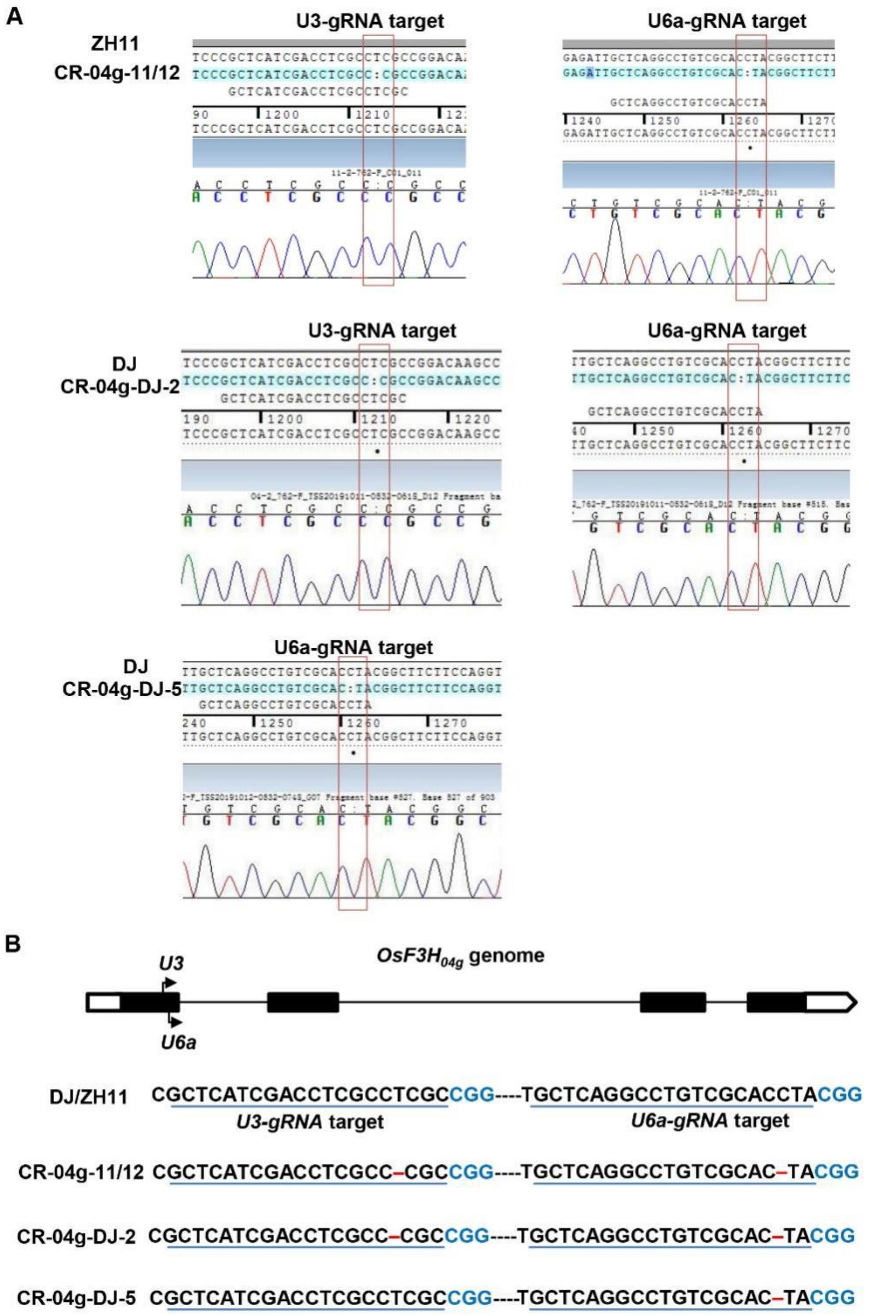

Figure S2. Identification of *OsF3H04g* gene editing rice lines. (A) The blast of *OsF3H04g* gene editing lines in *OsF3H04g* genome. CR-04g-11 and CR-04g-12 are in ZH11 background, CR-04g-DJ-2 and CR-04g-DJ-5 are in Dongjin (DJ) rice background. (B) Genotypes of the *OsF3H04g* gene editing lines in *OsF3H04g* genome. The horizontal line in red represents a base deletion and the gRNA target sequence is marked with blue underlines in *OsF3H04g* genome sequence.

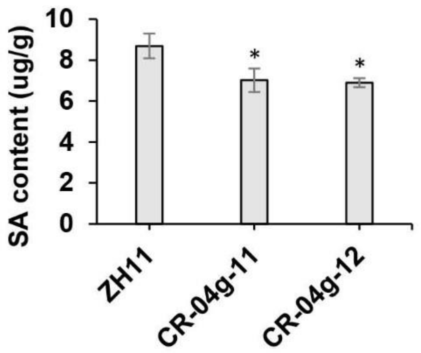

Figure S3. The salicylic acid (SA) content is decreased in *OsF3H04g* gene editing lines in ZH11 background. Data represent means ± SD, n=3. Asterisks mean significant differences between ZH11 and *OsF3H04g* gene editing lines (CR-04g-11 and CR-04g-12). (* $P \leq 0.01$, Student's *t*-test).

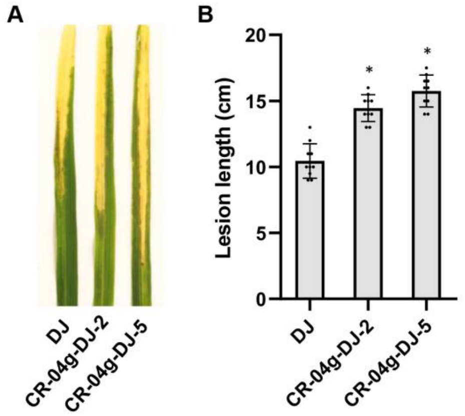

Figure S4. Gene editing of *OsF3H04g* increases rice susceptibility to bacterial leaf blight (BB) in DJ background. (A, B) Photograph of lesion expansions (A) and diagram of lesion lengths (B) in DJ and *OsF3H04g* gene editing lines (CR-04g-DJ-2 and CR-04g-DJ-5) caused by PXO99 at 14 days post inoculation (dpi). Data represent means ± SD, n=10. Asterisks mean significant differences between DJ and *OsF3H04g* gene editing lines. (* $P \leq 0.05$, Student's *t*-test).

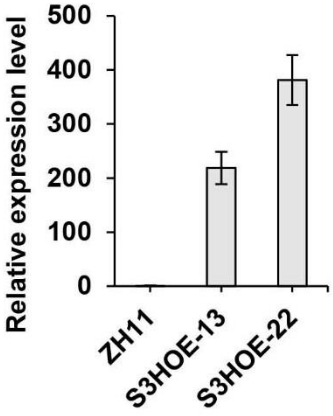

Figure S5. The expression level of *OsS3H* in the overexpression rice lines. *OsACTIN* was used as an internal control. Data represent means ± SD, n=3.

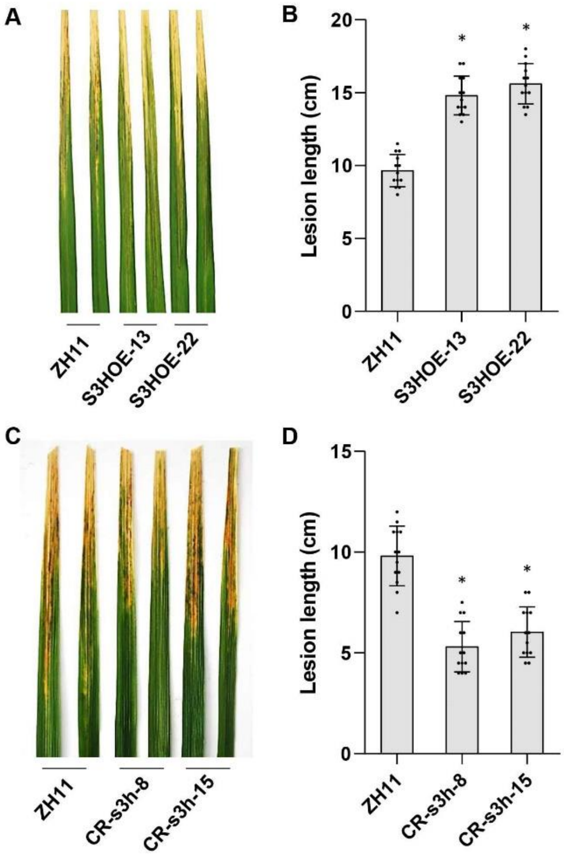

Figure S6. *OsS3H* negatively regulates rice resistance to BB. (A, B) Photograph of lesion expansions (A) and diagram of lesion lengths (B) in ZH11 and *OsS3H* overexpression lines (S3HOE-13 and S3HOE-22) caused by PXO99 at 14 dpi. (C, D) Image of lesion expansion (C) and diagram of lesion lengths (D) in ZH11 and *OsS3H* gene editing lines (CR-s3h-8 and CR-s3h-15) at 14 dpi with PXO99. Data represent means ± SD, n=13. Asterisks indicate significant differences between ZH11 and *OsS3H* overexpression or gene editing lines. (* $P \leq 0.05$, Student's *t*-test).

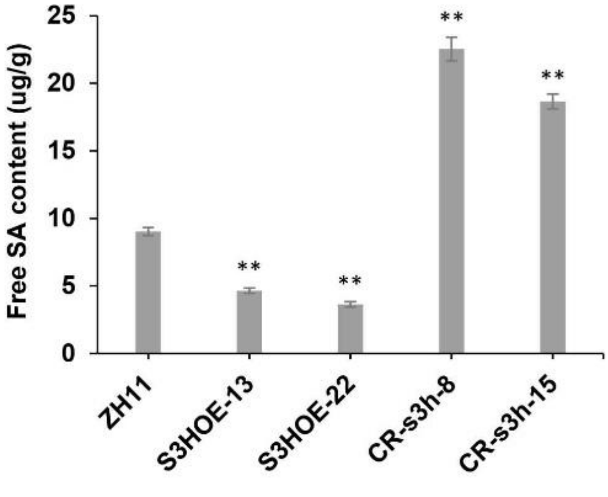

Figure S7. The contents of SA in *OsS3H* overexpression and gene editing lines in ZH11 background. Data represent means ± SD, n=3. Asterisks indicate significant differences between ZH11 and *OsS3H* transgenic lines. (** $P \leq 0.01$, Student's *t*-test).

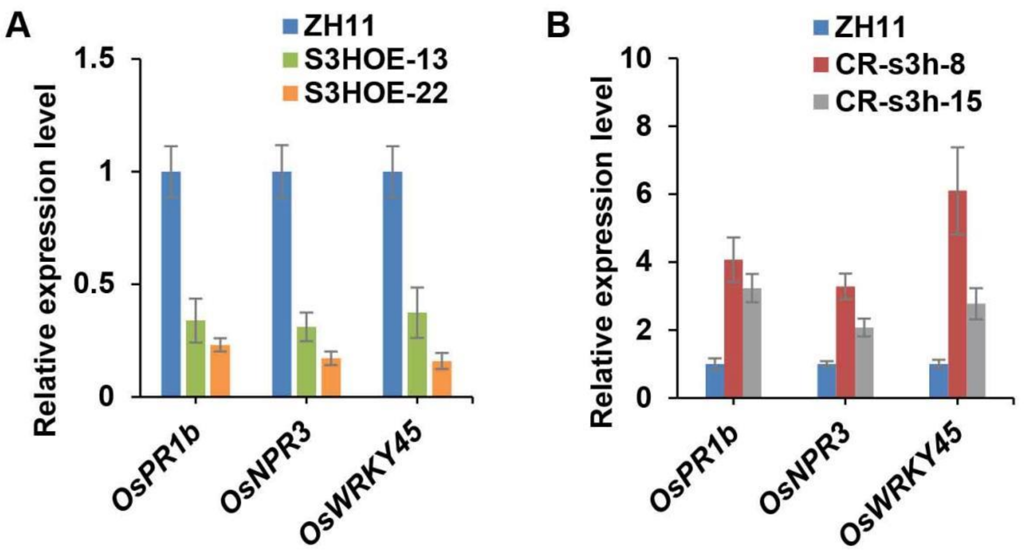

Figure S8. The expression level of *pathogenesis-related* (*PR*) genes in *OsS3H* overexpression and gene editing rice lines. (A, B) The relative expression level of *OsPR1b*, *OsNPR3* and *OsWRKY45* in ZH11, *OsS3H* overexpression lines (A) and gene editing rice lines (B). *OsACTIN* was used as an internal control. Data represent means ± SD, n=3.

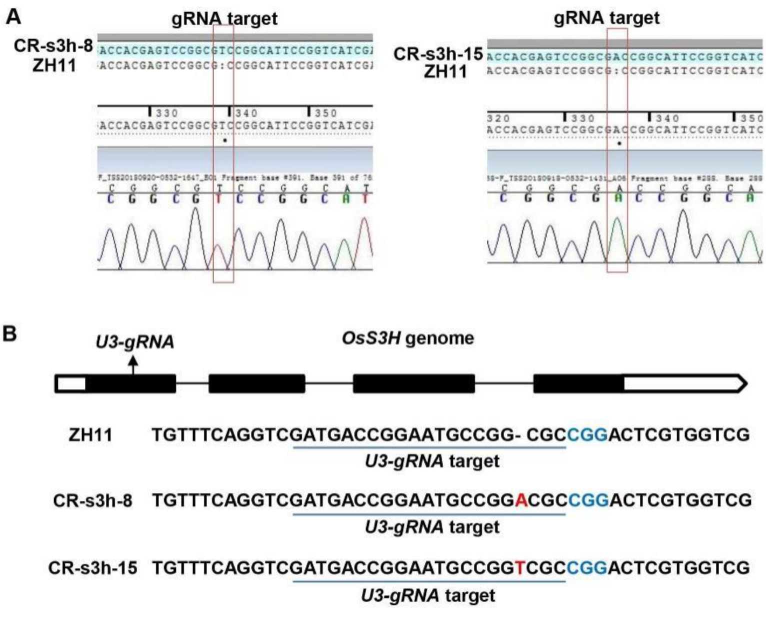

Figure S9. Identification of *OsS3H* gene editing rice lines. (A) The blast of *OsS3H* gene editing lines (CR-s3h-8 and CR-s3h-15) in *OsS3H* genome. (B) Genotypes of the *OsS3H* gene editing lines in *OsS3H* genome. The letters in red represent a base insertion and the gRNA target sequence is marked with blue underlines in the *OsS3H* genome sequence.

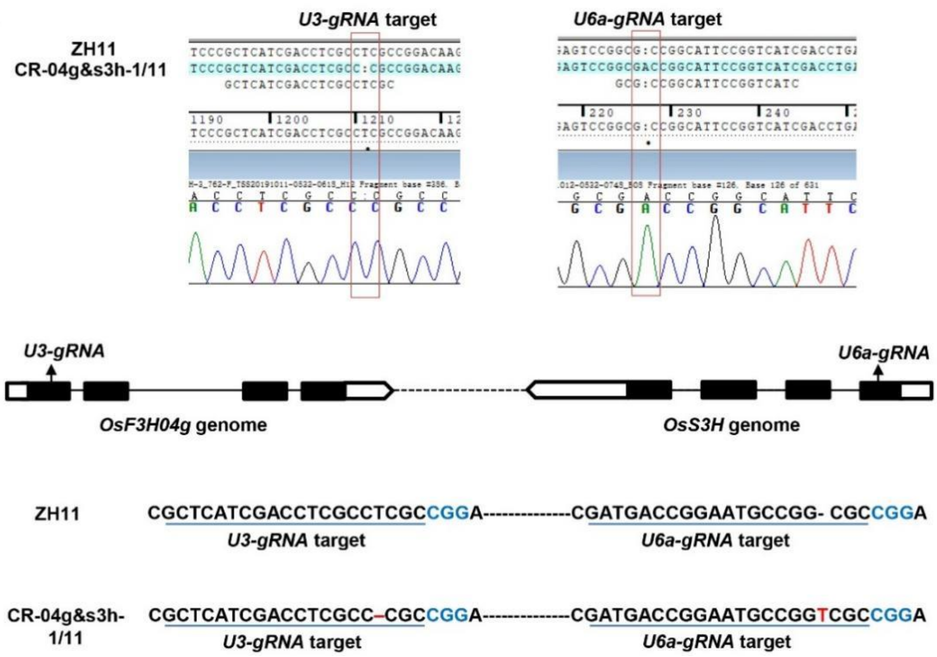

Figure S10. Identification of *OsF3H04g* and *OsS3H* double gene editing rice lines. (A) The blast of *OsF3H04g* and *OsS3H* double gene editing lines (CR-04g&s3h-1 and CR-04g&s3h-11) in *OsF3H04g* and *OsS3H* genomes. (B) Genotypes of the *OsF3H04g* and *OsS3H* double gene editing lines. The horizontal line in red represents a base deletion in *OsF3H04g* genome and the letter in red represents a base insertion in *OsS3H* genome. The gRNA target sequences are marked with blue underlines in *OsF3H04g* and *OsS3H* genome sequence.

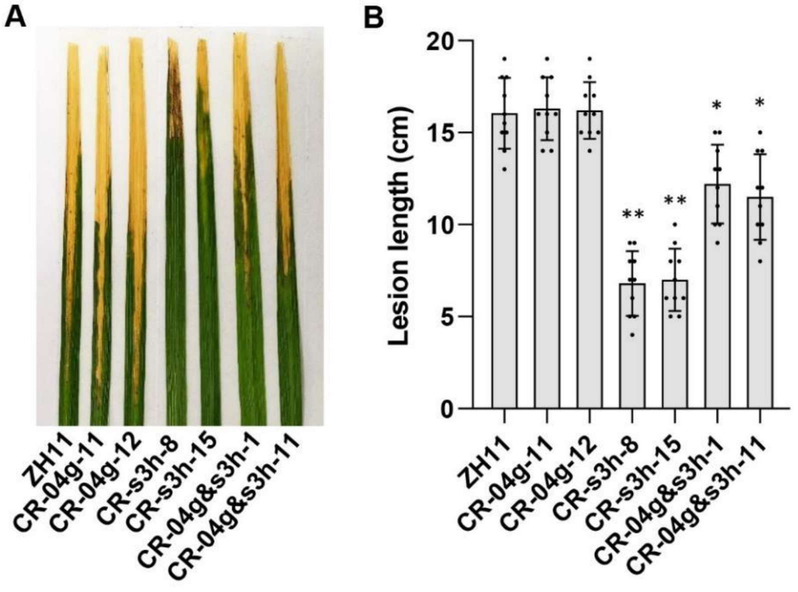

Figure S11. Gene editing both of *OsF3H04g* and *OsS3H* mildly increased rice resistance to BB. (A, B) Photograph of lesion expansions (A) and diagram of lesion lengths (B) in ZH11, *OsF3H04g* gene editing lines (CR-04g-11 and CR-04g-12), *OsS3H* gene editing lines (CR-s3h-8 and CR-s3h-15) and *OsF3H04g* and *OsS3H* double gene editing lines (CR-04g&s3h-1 and CR-04g&s3h-11) at 14 dpi with PXO99. Data represent means ± SD, n=10. Asterisks mean significant differences between ZH11 and gene editing lines. (* $P \leq 0.05$; ** $P \leq 0.01$, Student's *t*-test).
